# Mechanosensor for Proprioception Inspired by Ultrasensitive Trigger Hairs of Venus Flytrap

**DOI:** 10.34133/cbsystems.0065

**Published:** 2024-01-24

**Authors:** Qian Wang, Zezhong Lu, Deshan Wang, Kejun Wang

**Affiliations:** Jiangsu Provincial Key Laboratory of Advanced Robotics, Soochow University, Suzhou 215021, P.R. China.

## Abstract

Mechanosensors, as the core component of a proprioceptive system, can detect many types of mechanical signals in their surroundings, such as force signals, displacement signals, and vibration signals. It is understandable that the development of an all-new mechanosensory structure that can be widely used is highly desirable. This is because it can markedly improve the detection performance of mechanosensors. Coincidentally, in nature, optimized microscale trigger hairs of Venus flytrap are ingeniously used as a mechanosensory structure. These trigger hairs are utilized for tactile mechanosensilla to efficiently detect external mechanical stimuli. Biological trigger hair-based mechanosensilla offer an all-new bio-inspired strategy. This strategy utilizes the notch structure and variable stiffness to enhance the perceptual performance of mechanosensors. In this study, the structure–performance–application coupling relationship of trigger hair-based mechanosensors is explored through experiment and analysis. An artificial trigger hair-based mechanosensor is developed by mimicking the deformation properties of the Venus flytrap trigger hair. This bio-inspired mechanosensor shows excellent performance in terms of mechanical stability, response time, and sensitivity to mechanical signals.

## Introduction

Mechanosensors, as an indispensable part of exteroceptive and proprioceptive systems, are crucial for robots to perceive their external environments [1–5]. Compared with the tactile sensors (electronic skin), the mechanosensors protrude from the surface of objects to directly detect out-of-plane forces generated by the external environment [6–8]. In addition, mechanosensors with excellent characteristics, such as miniaturization, high precision, and portability, have attracted considerable research interest because they have an extremely important role in communication with the external environment [9]. In mechanosensors, the unique structure and the novel transduction material are the keys to optimize the performance of the sensors [10,11]. It is worth noting that the scattered mechanical signal cannot be directly detected by appropriate transduction elements such as piezoresistive materials, so the design of the sensor structure is of vital importance [12]. The cantilever beam structure, as the most widely used mechanical sensing structure of mechanosensors, greatly improves the sensing performance of the weak mechanical signal [13,14]. However, in contrast to the abundant research on the homogeneous cantilever-based sensing structure of tactile sensors, appropriate strategies for the development of variable stiffness cantilever beam structure used by tactile mechanosensors are scarce, which has become an obstacle for improving the performance of sensors to further optimize proprioceptive capability [15–18]. Here, developing an all-new and extraordinary sensing structure to overcome the performance limit of conventional mechanosensors is highly desirable.

Proprioceptive ability based on excellent mechanosensory structures are also important for plants, such as Venus flytrap [19–21]. Biological studies revealed that deflections of 2.9°, angular velocities of 3.4/s, and forces of 29 μN could produce action potential in the trigger hair of Venus flytrap, and the Venus flytrap can detect much smaller torques (160 nN·m) [22]. Our previous research on the elemental analysis of the same trigger hair revealed that basal podium had a low intensity of Ca compared to that of hair lever [23]. The difference of calcium content may cause the cantilever structure with variable stiffness. It is worth noting that sensory cells are longitudinal span notch structures that can make the sensory cells feel the external stimulus more easily [24]. In addition, with the help of a scanning electron microscope (SEM), the morphological structures of the trigger hairs were observed. We can clearly observe that the pipeline structure with thin walls between adjacent holes can greatly reduce the weight while ensuring its own flexural and torsional strength.

Here, we report a novel design and fabrication method for an artificial biomimetic trigger hair mechanoreceptor (BTHM) that consists of a rigid rod and a flexible base rod with a piezoresistor on the notch structure. The capillary glass tube with a certain diameter was used as the mold to prepare the sensor body, which can accurately control the diameter of the sensor body. Au film with a nano-crack structure was used as a piezoresistor deposited on the notch structure. Compared with other sensing methods, Au film with a nano-crack structure can better adapt to narrow structures and have higher sensitivity. In addition, the detection performance of the piezoresistor will be further improved under the effect of the notch structure. Besides, the artificial BTHM exhibited good sensitivity for mechanical signals, including minor load, vibrations, and air flow. In summary, we conducted a cross study on the sensing mechanism of trigger hair and the biomimetic manufacturing of sensors. The structure, elemental composition, and sensing method of trigger hair offer a novel strategy for sensor design. Building upon this approach and strategy, we have developed, fabricated, and analyzed the sensor, which have been successfully utilized for environmental detection.

## Materials and Methods

### The design and fabrication of biomimetic trigger hair mechanoreceptor

**Fig. 1.**
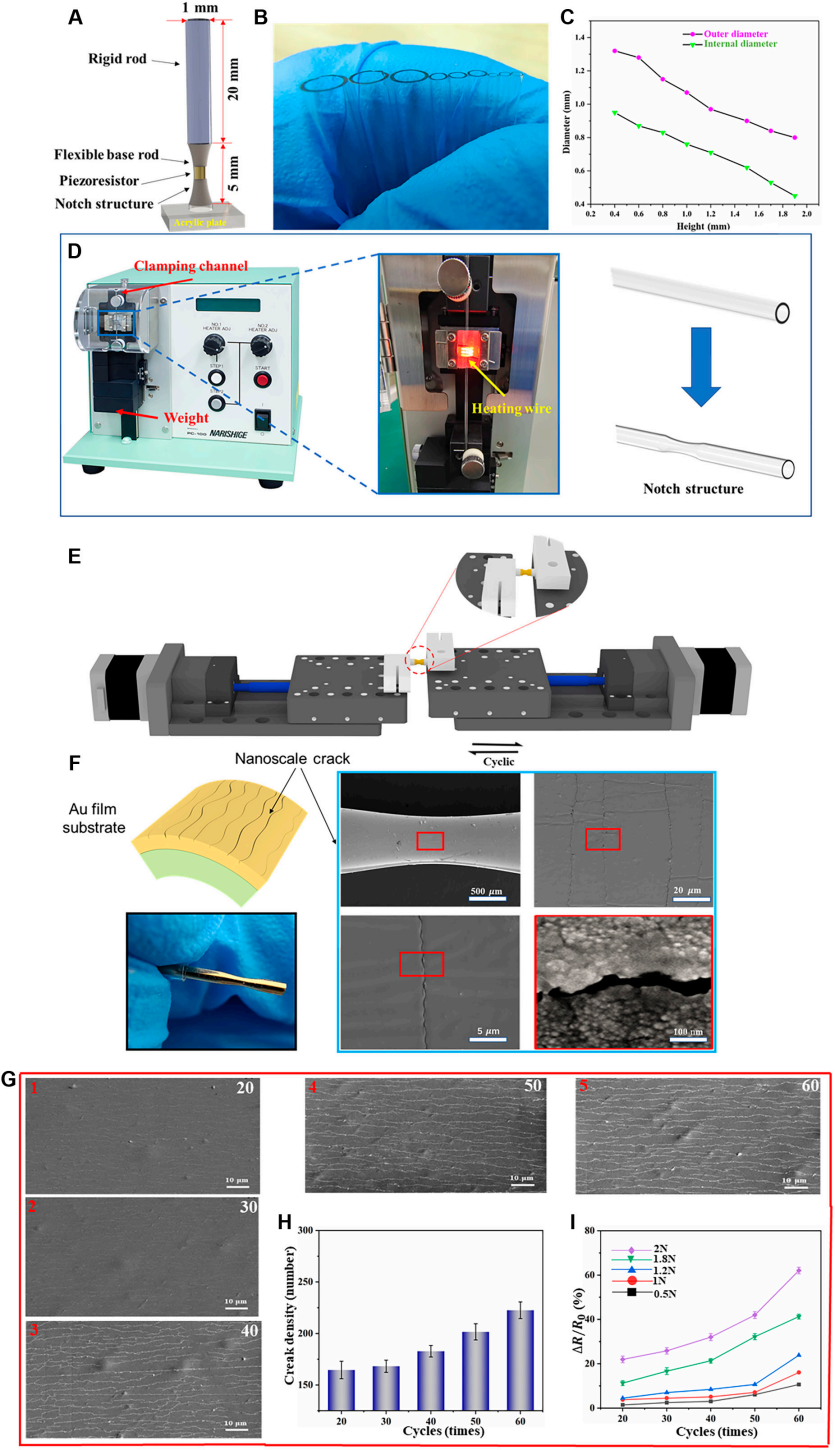
Design and fabrication of the BTHM. (A) Overall appearance of the BTHM. (B) Capillary glass tubes of different sizes can precisely control the rigid rod diameter. (C) Precise control of the inside and outside diameter of the notch structure of the capillary glass tube by the microelectrode puller. (D) The notch structure processing of the capillary glass tube. (E) Fabrication schematic diagram of the nanoscale crack. (F) Images of the piezoresistor with nanoscale crack. (G) SEM images with different crack densities after different number of cycles. (H) The influence of cycle times on crack density. (I) The response of ∆*R*/*R*_0_ to different forces.

The BTHM is a bio-inspired sensor that consists of a rigid rod and a flexible base rod with a piezoresistor on the notch structure, as shown in Fig. 1A. The design is based on the trigger hair of Venus flytrap, which was found to be a cantilever beam structure with variable stiffness. To mimic this structure, the BTHM employs a rigid–flexible coupling strategy, with a 20-mm-long rigid rod made of epoxy resin (elastic modulus of 2.415 GPa) and a 5-mm-long flexible base rod with a notch structure made of polydimethylsiloxane (PDMS) (elastic modulus of 0.0004 to 0.0035 GPa). The notch structure, which is present between the trigger hair's basal podium and hair lever, is where sensory cells span across. To replicate this, a notch structure is introduced at the base of the BTHM, and the piezoresistor is placed on it. The flexible base rod with the notch structure is 1 mm in diameter, and it is fixed on a 10 mm × 10 mm × 2 mm acrylic plate. The piezoresistor is made of Au film with nano-crack structures.

The fabrication process of BTHM is illustrated in Fig. 1. The rod and base of the BTHM are fabricated through a template method that utilizes capillary glass tubes as molds and the bottom substrate is created by laser ablation and acrylic substrate (Fig. 1B). The capillary glass tube was stretched using a microelectrode drawing instrument (PC-100, NARISHIGE, Japan) to obtain a base with a notch structure and different sizes of notch structures were produced by varying the drop height of weights (Fig. 1C and D and Movie S1). Commercially available PDMS (Sylgard 184; Dow Corning) was cast into the capillary glass tubes with notch structure to obtain a PDMS base with a notch structure. The PDMS solution was prepared by mixing monomer and curing agent at a weight ratio of 10:1. The mixture was then degassed in a vacuum chamber to eliminate any air bubbles before being poured into the capillary glass tubes. The solution was cured at 90 °C for 4 h, after which the solid PDMS structure was peeled off (Fig. S1).

The fabrication of the BTHM rods still involves the use of capillary glass tubes as molds. Prior to adding the curing material into the capillary glass tube, it is recommended to soak the inner wall of the tube with a release agent to form a thin film. Commercially available epoxy resin (Ultra clear crystal glue, Midisha) was cast into the capillary glass tubes to obtain the epoxy resin rod. To prepare the sensor rod, mix solutions A and B of the epoxy resin at a mass ratio of 3:1 and pour them into a container. Then, the mixture was stirred with a magnetic stirrer for approximately 8 min until it becomes uniform. Next, the container of the mixed solution was placed into a vacuum drying oven at room temperature and vacuum pressure was applied for about 10 min to remove any bubbles present in the mixture. The capillary glass tube was prepared by applying a layer of release agent on its inner surface. Then, the mixed solution was injected into the glass tube. Both ends of the mold were sealed and placed in the vacuum drying oven at 70 °C for curing for 1 h, allowing the resin to fully solidify. Once cured, the final rigid sensor rod was obtained by removing the capillary glass tube.

The piezoresistor is the core component of the bio-inspired mechanosensors. The piezoresistor in this study consists of 2 layers: the Au film with a thickness of 40 to 50 nm as the conductive layer and the Ge film with a thickness of 30 μm as the substrate layer. The ion sputtering instrument (E-1010, HITACHI, Japan) was utilized to perform the gold spraying process. To further improve the sensing performance, nanoscale cracks on the Au film were prepared. The nanoscale cracks were fabricated by using the traditional approach of mechanical bending of the flexible base rod. In this study, we developed a device to efficiently make the nanoscale crack (Fig. 1E). In the home-made device, the entire experiment was performed with a loading speed of 1 mm/s; the influence of the loading speed was effectively eliminated. Figure 1F clearly shows that the nanoscale crack patterns on the Au film were formed by bending the flexible base rod under repeated loading and unloading. Therefore, the crack density can be adjusted through changing the number of cycles. Figure 1H and I shows the influence of the number of cycles on crack density and detection performance. Figure 1G and H shows that the number of cracks increases from ≈165 to 225 as the number of cycles increases from 20 to 60. In addition, the more cycles performed, the stronger the detection ability for the load (Fig. 1I).

Thus far, we have separately considered the design and preparation of the rigid rod, flexible base rod, and the piezoresistor. Next, assemble those parts together. The rigid rod and the flexible base rod were bonded together by using quick-drying adhesive. Then, silver wires with diameters of 0.05 mm and 1.5 mm were bonded to the upper and lower ends of the base conductive layer with liquid metal separately. Finally, the sensor was fixed on the acrylic plate to complete the preparation of the sensor.

### Experimental characterization

To enhance the observation of the trigger hairs of Venus flytrap and the nanoscale cracks on the Au film, we utilized the optical microscope (OM; VHX-7000, KEYENCE, Japan) and the SEM (EVO 18, ZEISS, Germany).

To enable systematic analysis of BTHM performances, a testing device was constructed with a modal exciter (SA-JZ050,China), a signal generator, a digital multimeter (Keysight34465A, America), a vibration isolation platform (MEIRITZ,Japan), and a fixed bracket. This setup aimed to optimize the sensing performances of the BTHM by measuring their parameters accurately. The BTHM base is mounted on a bracket and the exciter is fixed on a vibration isolation platform. A digital multimeter is connected to the positive and negative terminals of the sensing element. The signal generator sends pulse signals at varying frequencies to the exciter, which, in turn, converts electrical signals into displacement signals that act on the BTHM's rod. As a result, the BTHM undergoes slight angular deflection, leading to corresponding resistance changes in the base. The digital multimeter then measures and displays the resistance change on a liquid crystal display screen (Fig. S2).

In order to verify the detection ability of BTHM, we carried out the micro-load, micro-airflow, and vibration experiment of BTHM respectively. For the micro-load detection experiment, 6 different quality aluminum and silver wire segments in the range of 0.0025 g to 0.0069 g served as loading loads. Those wire segments were released from a height of 2 cm away from the BTHM rod. For the micro-airflow detection experiment, the resistance changes of BTHM caused by continuous airflow and single airflow impact were studied. For the vibration detection experiment, we used various vibration sources such as ball dropping from different heights and finger tapping on a desktop.

During the stress test, a vibrator is still used to apply pressure signals, with the frequency of the vibrator set at 0.5 Hz. The BTHM is fixed on a bracket, and the top end of the rod is in contact with the top rod of the vibrator. In this case, the bending stress caused by the deflection of the sensor rod at the base can be well detected.

## Results and Discussion

### Biomimetic trigger hair mechanoreceptor inspired by Venus flytrap

**Fig. 2.**
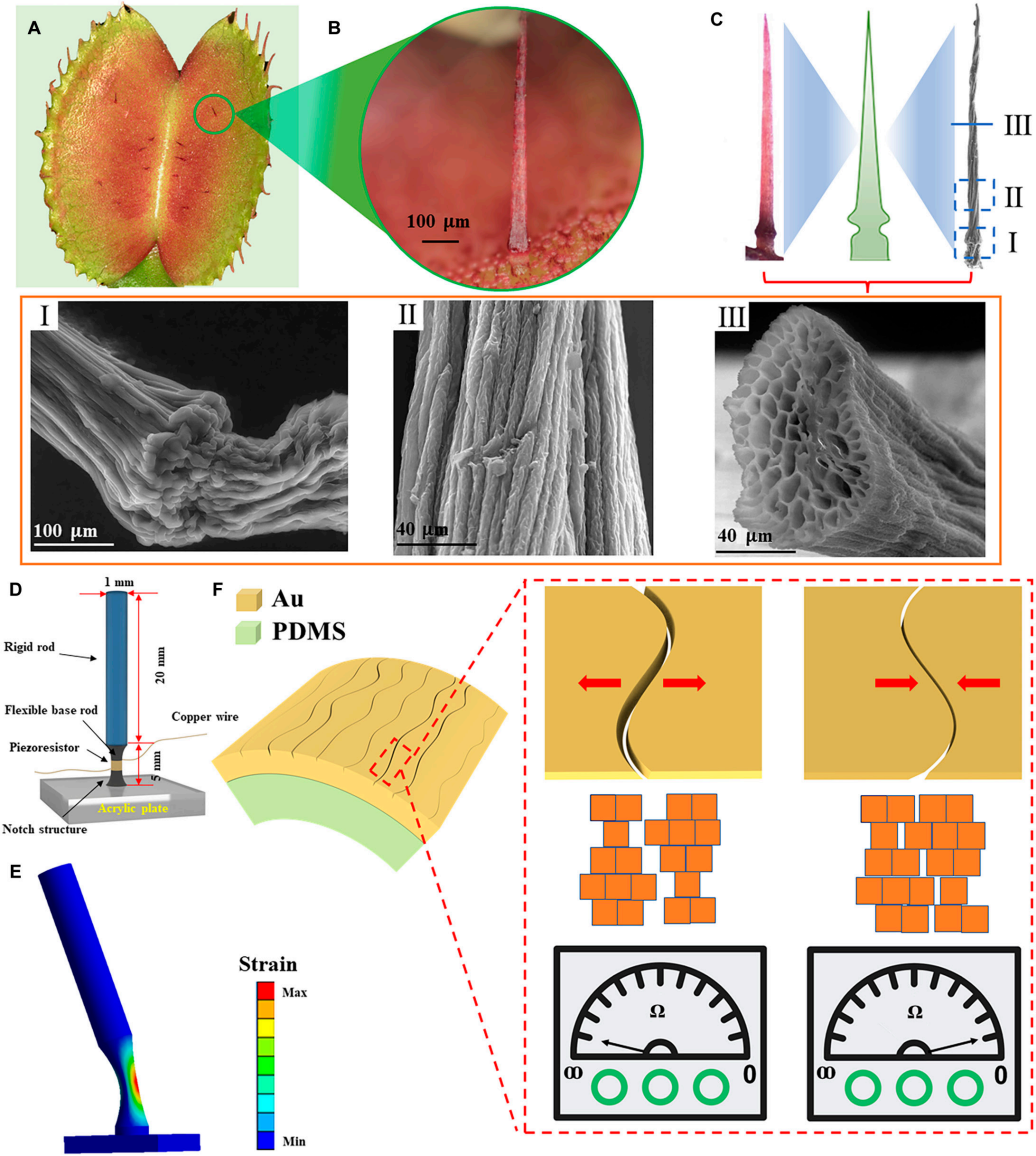
Morphological characteristics of the Venus flytrap trigger hair and the principle of the artificial trigger hair-based mechanosensor. (A to C) Morphological characteristics of the trigger hair, which is a special cantilever beam structure. Positions I and II display magnified images of the basal podium and hair lever of the Venus flytrap trigger hair, respectively, as observed through SEM. Position III is the cross-sectional view of the hair lever of the Venus flytrap trigger hair observed through SEM. (D) Schematic illustration of the bio-inspired trigger hair-based mechanosensor. (E) Finite element simulation of the BTHM under external mechanical stimuli. (F) Schematic diagram of the composition and operating principle of the piezoresistor.

The Venus flytrap captures prey by sensing the insects that touch its tactile receptors, known as trigger hairs. Numerous studies have demonstrated that the ultra-sensitive tactile perception of almost all types of mechanosensilla is primarily attributed to the unique mechanical properties of their functional structures, which can effectively detect external signals and convert them into mechanical reactions [24–27]. The Venus flytrap's ultra-sensitive perception relies on 3 or 4 trigger hairs with a special cantilever beam structure located on its leaves (Fig. 2A). The morphological and structural characteristics of the trigger hair-based mechanosensilla of Venus flytrap are presented in Fig. 2A to C. The results show that the trigger hair is a special cantilever beam structure with large longitudinal and radial proportion, and the radial size increases gradually from top to bottom (Fig. 2B). It is composed of a hair lever and a basal podium, and there is a notch structure near the hair base (Fig. 2C, I and II). Meanwhile, our previous studies have shown that though on the same trigger hair, basal podium had a low intensity of Ca compared to that of hair lever, indicating the differences in material properties [21]. Building on our previous research, we performed a touch experiment on the trigger hair of Venus flytrap by gently applying force to the hair lever. It was evident that the hair lever of the trigger hair was deflected, and the basal podium was curved by observing the trigger hair under the OM (VHX-7000, KEYENCE, Japan), providing further evidence for the difference in material properties between the hair lever and the basal podium (Movie S2). To have a deeper discussion on the difference of mechanical characteristics between variable stiffness cantilever beam structure and homogeneous cantilever beam structure, a model was established and analyzed through finite element method. By conducting finite element analysis on a custom cantilever beam model, the results show that the base of the cantilever beam model is the concentration area of strain rather than the rod part (Fig. 2E). In addition, the internal structure of the trigger hair was revealed by SEM, providing evidence for further investigation into the high-sensitivity sensing mechanism of trigger hairs and offering insights for the development of high-performance sensors. The cross-section of the trigger hair rod, as illustrated in Fig. 2C, III, exhibits a distinctive pipeline structure consisting of numerous small holes arranged in a honeycomb-like fashion. Specifically, the hole extends longitudinally to form a structure similar to a conduit, which can reduce its own weight to enhance stability.

Inspired by the special cantilever beam structure of the trigger hair, we have developed a mechanosensor that exhibits extremely high sensitivity to various mechanical signals such as stress, pressure, vibration, and displacement, as shown in Fig. 2D to F. The BTHM mainly consists of a rigid rod and a flexible base rod with a piezoresistor on the notch structure (Fig. 2D). Similar to the mechanosensilla based on biological trigger hairs, the artificial mechanosensors employ a variable stiffness cantilever beam structure that can readily undergo deformation in response to mechanical signals, as depicted in Fig. 2E. The piezoresistor is composed of a layer of Au film based on a nanoscale crack structure. Its ultrahigh sensitivity is attributed to the drastic change in resistance. When subjected to external mechanical stimulation, the resistance will undergo a sudden jump from a small value to infinity with the disconnection–reconnection process of nanoscale jagged crack edges (Fig. 2F). Combined with the extremely deformable characteristics of the variable stiffness cantilever beam structure under a tiny mechanical signal, our bio-inspired mechanosensor showed excellent sensing performance for various mechanical signals.

### Performance characterization of biomimetic trigger hair mechanoreceptor

#### Fatigue and frequency testing of biomimetic trigger hair mechanoreceptor

**Fig. 3.**
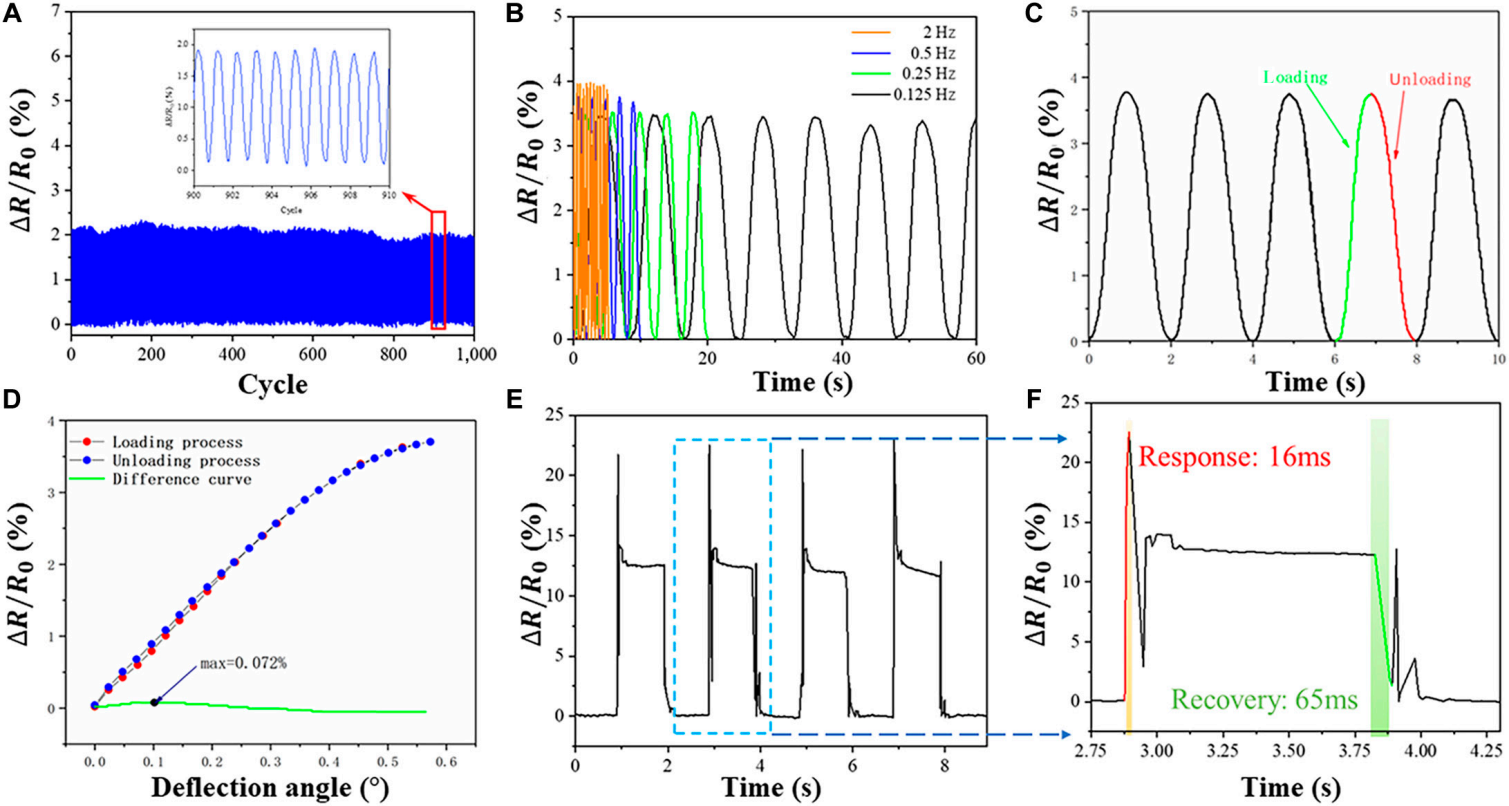
Performance characterization of BTHM. (A) Relative resistance change–time curves of the BTHM for 1,000 load/unload cycles. (B) Response of ∆*R*/*R*_0_ under load/unload cycles at a frequency from 0.125 to 2 Hz. (C) Relative resistance change–time curves of the BTHM for load/unload processes. (D) Analysis curve and difference curve between loading and unloading processes. (E and F) Fast response and recovery time of the BTHM.

To evaluate the durability and stability of BTHM, it is necessary to conduct a fatigue test. This test will determine if BTHM can maintain its stability by measuring the relative change in resistance after multiple cyclic loading. Passing this test would prove that BTHM has good durability. BTHM with a nano-microcrack structure, featuring a base length of 5 mm and a rod length of 20 mm, underwent 1,000 cyclic loading tests. The fatigue test experiments were conducted by continuously applying cyclic displacement signals using a vibrator, with the frequency of the vibrator set to 2 Hz and the loading displacement set to 0.15 mm. Figure 3A shows that the resistance signals of BTHM exhibit strong periodicity, with consistent peak amplitudes throughout the 1,000 cyclic loading tests. The biomimetic hair sensor reported by Huang et al. [28] also underwent 1,000 fatigue cycle tests and the stability remains excellent. This remarkable stability of BTHM paves the way for its future use in detection applications.

The response behavior of BTHM is significantly influenced by loading frequency, which is an important indicator for measuring its performance. To determine this effect, the BTHM with a nano microcrack structure (base length of 5 mm and rod length of 20 mm) underwent loading tests at different frequencies using a vibration exciter. The output displacement of the vibration exciter was set to 0.15 mm. Results showed that in the loading frequency range of 0.125 to 2 Hz, the response of BTHM was stable (Fig. 3B).

### Hysteresis and response test of biomimetic trigger hair mechanoreceptor

Measuring the level of hysteresis is also a crucial indicator for evaluating the performance of strain sensors. To obtain the resistance change curve of the BTHM, it is necessary to load and unload the BTHM multiple times using an exciter and record its corresponding resistance changes. The loading process using the exciter involves a maximum deflection angle of 0.7° for the BTHM rod and a loading frequency of 0.5 Hz. Experimental curves for loading and unloading are as shown in Fig. 3C.

Figure 3D shows evidence that the loading and unloading curves of BTHM almost overlap. The maximum difference between the 2 curves is only 0.072%.

To quantitatively compare the degree of hysteresis, the following definition is proposed:

$$\mathrm{DH}=\frac{A_{S}-A_{R}}{A_{S}}\times 100\%$$ (1)

Here, *A_S_* and *A_R_* represent the area under the curve during the deflection and recovery phases of BTHM, respectively.

The DH value exhibits a linear relationship with hysteresis. A smaller DH value indicates a lower level of hysteresis. Based on the calculated DH values of 0.29%, it can be concluded that BTHM exhibits very low levels of hysteresis.

Next, the response performance of BTHM was tested using a square wave pulse signal with a frequency of 0.5 Hz and a maximum displacement of 0.3 mm. Figure 3E shows the corresponding response curve. The response time and recovery time provide an indication of BTHM's response speed to changes in measurement. A shorter response and recovery time indicates faster response speed and less delay caused to the entire device.

The sharp peaks observed at both ends of the response curve in Fig. 3F are attributed to the large inertia caused by instantaneous loading and unloading of the pulse signal, particularly during the moment of pulse loading. The fast response speed and recovery speed of BTHM are reflected by the short response time of approximately 16 ms, while the recovery time is slightly longer than the response time, taking about 65 ms. It is understandable that during the recovery process, the slight oscillation of the rod due to the flexibility of the base prevents it from immediately returning to its original position, resulting in a longer recovery time compared to the response time.

The response time of the sensor in this study, which is 16 ms, is better than the 43 ms and 17.5 ms reported by Zhang et al. [29] and Zhou et al. [30], respectively.

#### Sensitivity test of biomimetic trigger hair mechanoreceptor

**Fig. 4.**
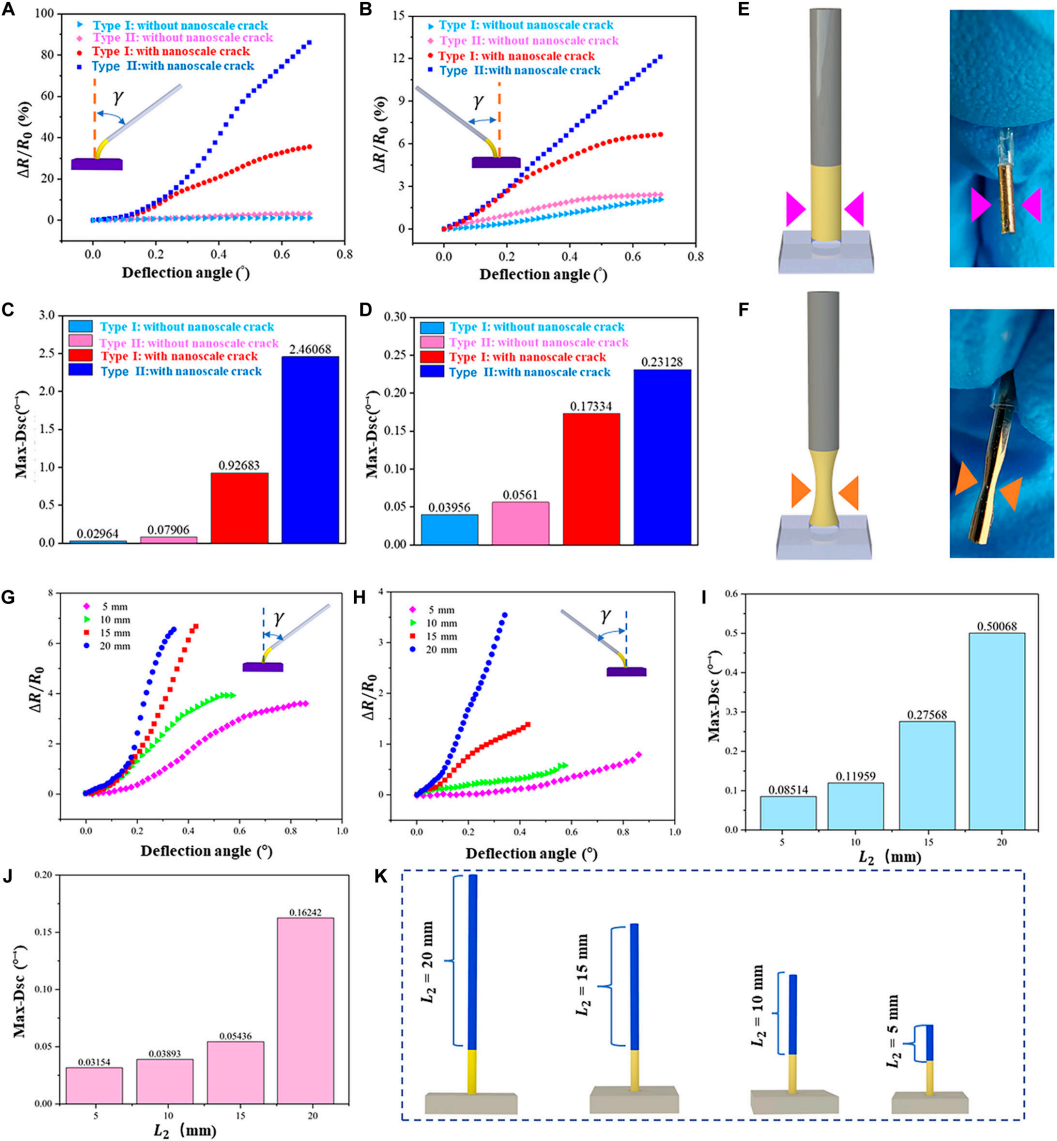
Sensitivity test of the BTHM. (A and B) Relative resistance change–deflection angle curves of the BTHM under tensile/compressive stress. (C and D) Column diagram of maximum sensitivity of the BTHM under tension/compression stress. (E and F) Images of base rod smooth and notch structure. (G and H) Curves of relative resistance change–deflection angle of the BTHM with 4 different lengths of rigid rod under tensile/compressive stress. (I and J) Column diagram of maximum sensitivity of the BTHM with 4 different lengths of rigid rod under tensile/compressive. (K) Schematic diagram of the BTHM with different lengths of rigid rod.

The influence of the base rod notch structure and nanoscale crack structure on sensitivity was investigated. Two control experiments were conducted on a base rod with a smooth base rod (Fig. 4E) and a notch structure (Fig. 4F), respectively, both having nano cracks on the surface. These are identified as type I and type II, respectively. The specific test parameters are presented in Table. Furthermore, the exciter is configured to operate at a frequency of 0.5 Hz.

**Table.** Structural parameters of sensing elements to be tested

Number	Type	Length of base (mm)	Length of rod (mm)	Nano-crack structure
1	I	5	20	Yes
2	I	5	20	No
3	II	5	20	Yes
4	II	5	20	No

To quantitatively assess the sensitivity of BTHM, we introduce the deflection sensitivity coefficient (Dsc) value, which is defined as follows:

$$\mathrm{Dsc}=\left(∆R/R_{0}\right)/\gamma \;$$ (2)

where *R*_0_ is the initial resistance, Δ*R* = *R* − *R*_0_ represents the relative change in resistance of BTHM, and γ represents the deflection angle of BTHM caused by the top rod of the exciter. For small deflection angles, it can be approximated using the following formula:

$$\gamma =\text{arcsin}\left(l/H\right)\;$$ (3)

In Eq. 3, *l* stands for the displacement output of the exciter's top rod, and *H* refers to the distance between the point where the top rod applies force on the BTHM and its base.

As depicted in Fig. 4A and B, the response curve indicates that both type I and type II sensors can detect small deflection angles under tension and compression stresses and generate corresponding resistance changes, irrespective of whether the conductive layer of BTHM has a nano-crack structure or not.

Under tensile stress, when considering the type II sensor, it can be observed that at the same maximum deflection angle of 0.7°, the relative resistance change (∆*R*/*R*_0_) of the sensor without a nano-crack structure is 3.164, whereas that of the sensor with a nano-crack structure is 86.096, which is more than 27 times greater than the former as shown in Fig. 4A. In addition, the maximum Dsc values of the sensor with a nano-crack structure (2.46068°^−1^) is approximately 31 times larger than that of the sensor without a nano-crack structure (0.07906°^−1^) as illustrated in Fig. 4C. It should also be noted that the type II sensor with a nano-crack structure exhibits only 12.106 ∆*R*/*R*_0_ at 0.7° deflection, and the maximum Dsc values within the same range is only 0.23128°^−1^, which are significantly lower than the relative parameters under tensile stress (as shown in Fig. 4B to D). However, there is no significant difference between the∆*R*/*R*_0_ and maximum Dsc values of the type II sensor without the nano-crack structure under both tensile and compressive stresses. Then, the effect of rod length on BTHM sensitivity was discussed. While keeping the base length constant at 5 mm, BTHMs with nano-crack structures that had rod lengths (L2) of 5 mm, 10 mm, 15 mm, and 20 mm were tested, as depicted in Fig. 4K. Due to the varying lengths of the BTHM rods, the maximum deflection angle changes under the same exciter output parameters. Specifically, a shorter rod results in a greater deflection angle. As shown in Fig. 4G and H, it is evident that under both tensile and compressive stress states, the relative change in resistance increases with an increase in the rod length for the same deflection angle. The maximum Dsc values for rod lengths of 5 mm, 10 mm, 15 mm, and 20 mm under tensile stress are 0.08514°^−1^, 0.11959°^−1^, 0.27568°^−1^, and 0.5068°^−1^, respectively. Conversely, the maximum Dsc values under compressive stress are 0.03154°^−1^, 0.03893°^−1^, 0.05436°^−1^, and 0.16242°^−1^, respectively. It can be observed that the longer the rod length, the larger the maximum Dsc values of the sensor. Additionally, the maximum Dsc values of the sensors with different rod lengths under tensile stress are greater than those under compressive stress.

### The detection capability of biomimetic trigger hair mechanoreceptor

#### Load detection characterization of biomimetic trigger hair mechanoreceptor

**Fig. 5.**
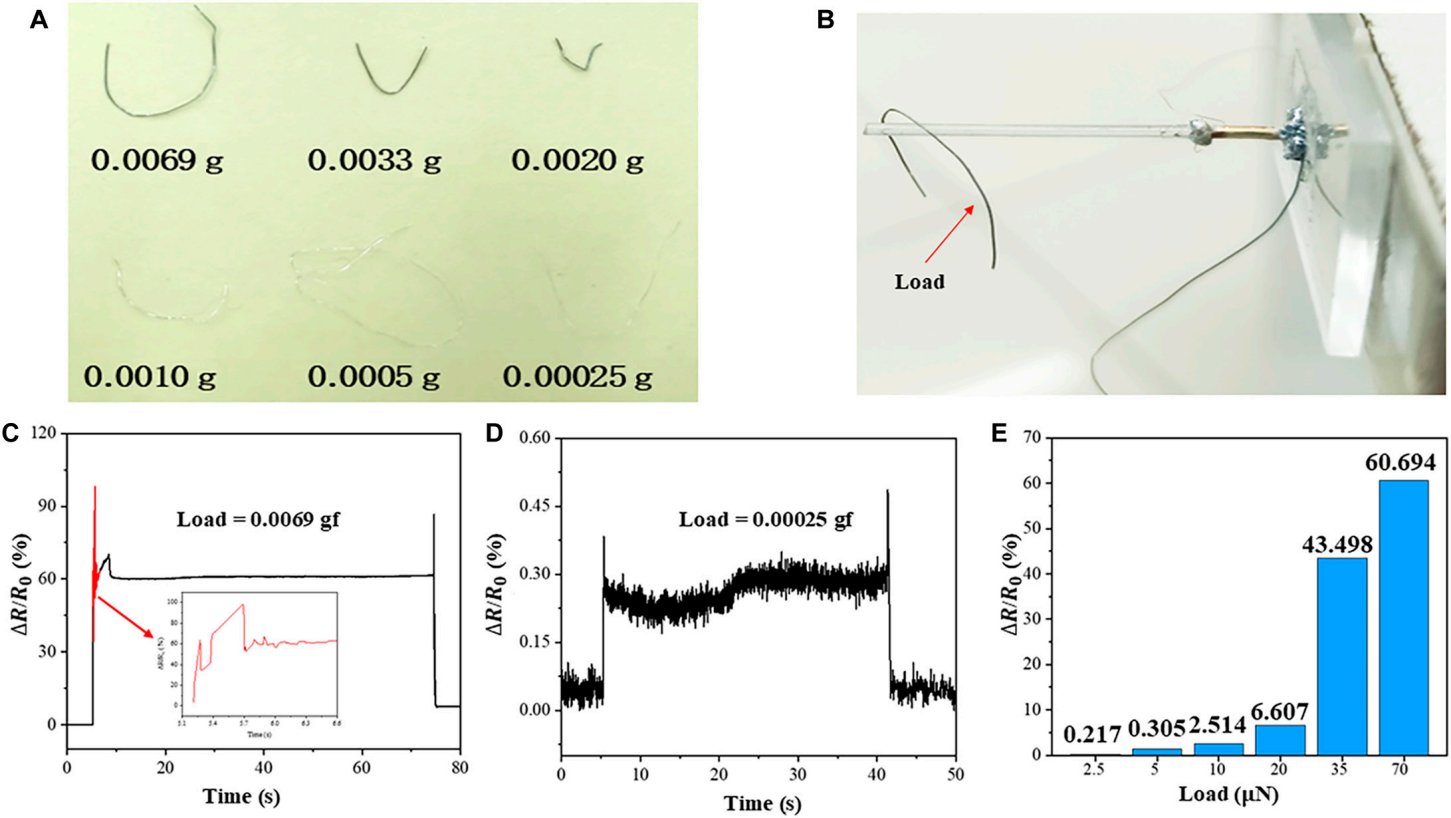
Load detection of BTHM. (A) Images of 6 different quality grades of wire. (B) Loading diagram of the 0.0069 g aluminum wire. (C and D) Response curves of ∆*R*/*R*_0_ when the load is 0.0069 gf aluminum wire and 0.00025 gf silver wire, respectively. (E) Comparative analysis of the change rate of resistance corresponding to different loading loads.

To evaluate the sensitivity of BTHM in detecting small loads, we conducted an experiment using 6 segments of aluminum and silver wire of varying masses ranges from 0.00025 g to 0.0069 g as loading loads (Fig. 5A and B). The wires were released from a height of 2 mm above the top of the rod (as shown in Fig. 5C). The results show that BTHM can easily detect resistance changes caused by as little as 69 μN load. Specifically, the aluminum wire with a mass of 0.0069 g fell on the BTHM rod and became stable after slight shaking for 0.3 s. The response curve showed protruded peaks at both ends, which were caused by the inertia of loading and unloading the aluminum wire. The corresponding ∆*R*/*R*_0_ remained stable at around 60% (Fig. 5D). On the other hand, BTHM was able to detect weak loads applied to the silver line, as low as 0.00025 gf (2.5 μN). However, the resistance response curve fluctuated and only showed about 0.3% ∆*R*/*R*_0_ as shown in Fig. 5E. Generally, ∆*R*/*R*_0_ was directly proportional to the loading load of BTHM. Overall, it is important to note that the ability of BTHM to detect small loads is highly dependent on the geometric properties of the object being loaded. Nonetheless, our findings suggest that BTHM has promising potential for various applications, especially those requiring high sensitivity in detecting small loads.

#### Air flow detection of biomimetic trigger hair mechanoreceptor

**Fig. 6.**
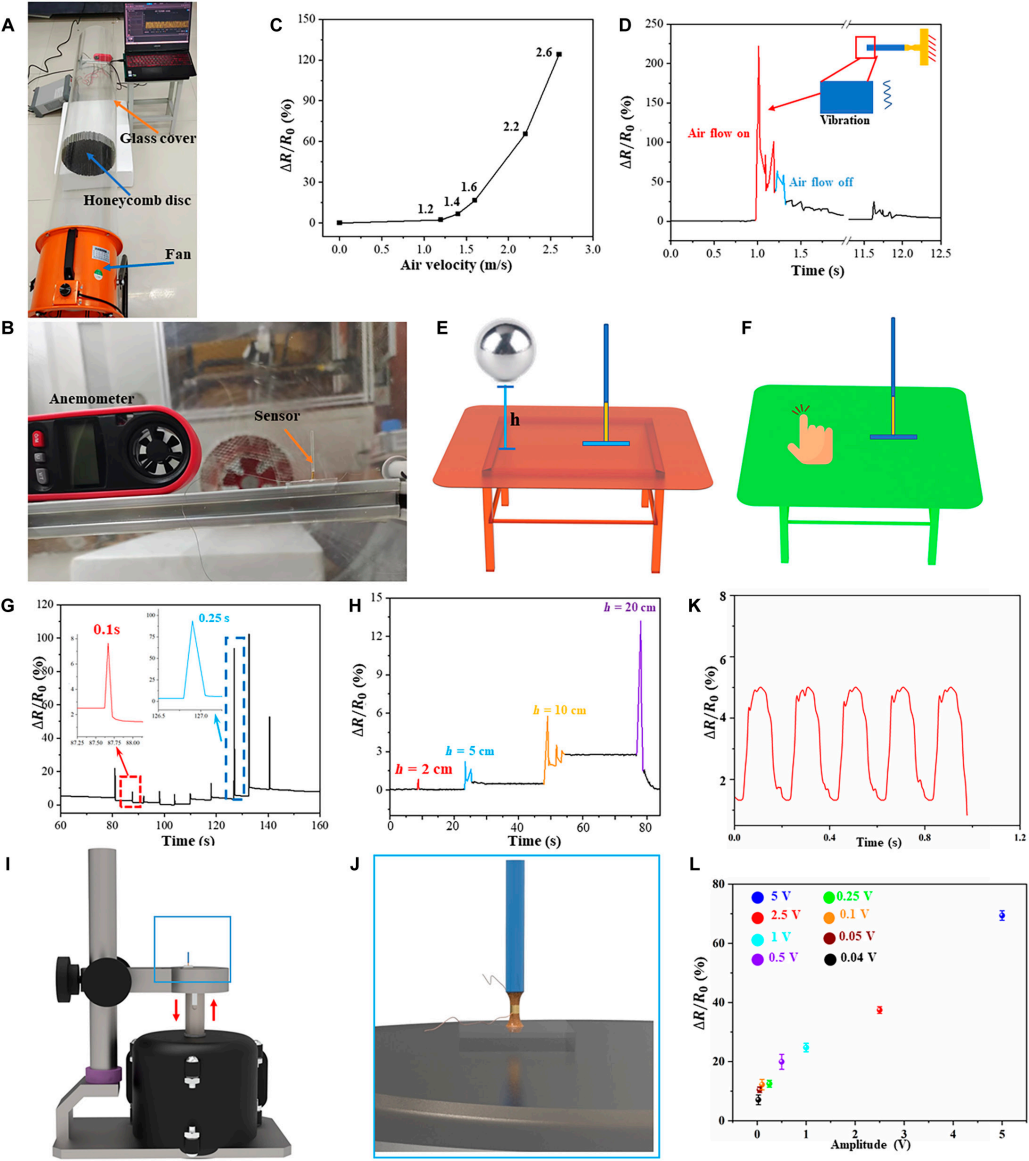
Air flow and vibration detection of BTHM. (A) Home-made experimental device used to characterize the air velocity detection performance. (B) Front view of the home-made experimental device used to characterize the air velocity detection performance. (C) Response curves of ∆*R*/*R*_0_ to the change in air velocity. (D) Relative resistance change–time curves of the BTHM when subjected to single air impact. (E and F) Schematic model diagram of vibration detection. (G) Relative resistance change–time curves of the BTHM when subjected to the vibration caused by finger tapping on the table. (H) Relative resistance change–time curves of the BTHM when subjected to the vibration caused by different falling heights of the steel ball. (I) Schematic diagram of vibration experiment testing. (J) Magnified diagram of the fixed position of the sensor. (K) The maximum frequency that the sensor can detect is 5 Hz. (L) The response of the sensor resistance change rate caused by different amplitudes of the shaker.

To evaluate the response of BTHM to air flow, we developed a home-made system consisting of a fan, honeycomb disk, and hood (Fig. 6A). The fan provided air flow of varying intensities, which flowed through the honeycomb disc along the internal duct of the hood. The honeycomb disc rectified the air flow, and collected and regulated the scattered air flow generated by the fan. The BTHM was fixed on a bracket at the outlet of the wind hood and an anemometer was attached to measure the wind speed flowing through the BTHM in real time as depicted in Fig. 6B. Figure 6B shows that ∆*R*/*R*_0_ of BTHM increased with the increase of wind speed. At a wind speed of 1.2 m/s, the ∆*R*/*R*_0_ of the BTHM was only about 2.3%. However, when the wind speed increased to 2.6 m/s, the ∆*R*/*R*_0_ increased up to about 124.3%, showing a nonlinear relationship with wind speed (Fig. 6C). Further study of the BTHM response under single air flow impact revealed that ∆*R*/*R*_0_ increased from 0 to about 221.8% in a very short time after the air flow impact. Although the relative change of the resistance of the BTHM did not immediately return to the baseline position after the air flow impact, it did fluctuate due to the vibration caused by the air flow impact as shown in the red curve part of Fig. 6D. Moreover, Fig. 6D also shows that the BTHM can even detect the impact caused by the fluid inertia resulting from the sudden interruption of the air flow as evident in the blue curve part. Therefore, the resistance response of the BTHM can help determine different air flow states in the surrounding environment.

#### Vibration detection of biomimetic trigger hair mechanoreceptor

Detecting external vibration signals is a crucial application scenario for BTHM. Figure 6E demonstrates the experimental setup where the BTHM is fixed on the table's side, and a small steel ball is dropped from different heights in the same vertical direction. Figure 6K shows that the resistance change rate (∆*R*/*R*_0_) increases from 0.9% to 13% as the drop height of the ball increases from 2 cm to 20 cm. The resistance change rate increases with the ball's drop height, and the BTHM accurately detects when the ball rebounds after exceeding a specific falling height. When the ball is dropped from a height of 5 cm and 10 cm, there are 2 or 3 peaks of ∆*R*/*R*_0_, respectively, which decrease successively, indicating that the ball bounces back once or twice after the initial landing. The attenuation of the peak value is due to the damping vibration process when the ball bounces back after landing (Fig. 6H). The effect of vibration intensity on the BTHM's resistance response was also analyzed by knocking the sensor's same position with different forces, as shown in Fig. 6F. A weaker knocking force (red rectangular area) results in a ∆*R*/*R*_0_ peak value of about 7.8%, with a response time of approximately 0.1s for returning to baseline (Fig. 6I). With a stronger knocking force (blue rectangular area), the ∆*R*/*R*_0_ peak value reaches about 92%, but the response time for returning to baseline is extended to approximately 0.25s (Fig. 6J). Further, we conducted an experiment with the help of a support bracket and a shaker to explore the frequency and amplitude of the sensing sensor (Fig. 6I to L). When the frequency of the shaker was greater than 5 Hz, the signal perceived by the sensor became noticeably chaotic (Fig. 6K). Therefore, the maximum frequency limit that the sensor can accept is 5 Hz. Similarly, when the amplitude is less than 0.5 V, the response of the sensor is also in a weak state (Fig. 6L).

### Application in environmental detection of BTHM

**Fig. 7.**
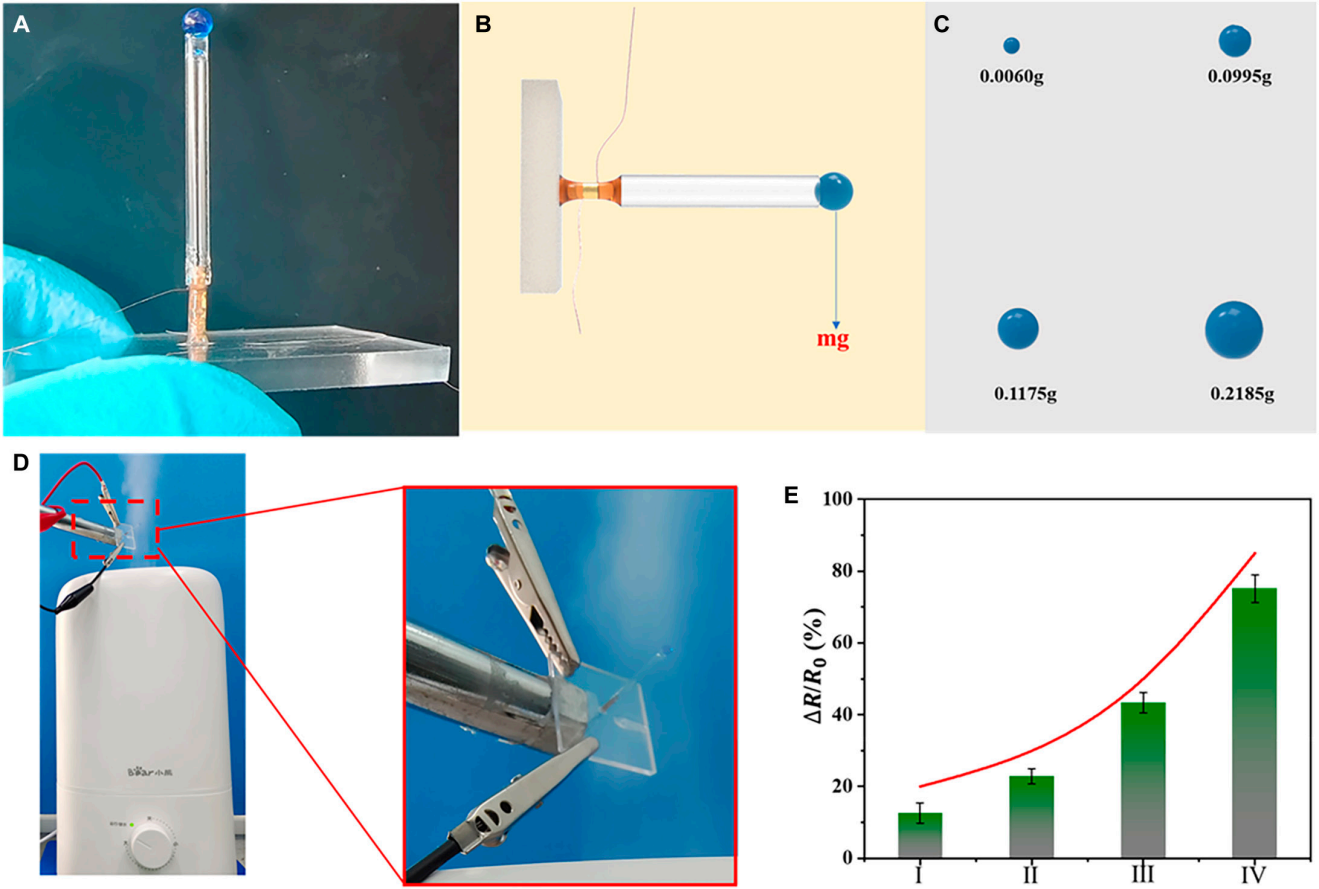
(A) Schematic diagram of the combination of the superabsorbent polymer and BTHM. (B) The schematic diagram shows the response of a sensor to the change in gravitational force caused by the absorption of water by a hydrogel (C) Schematic diagram of the huge mass change range of the superabsorbent polymer. (D) Humidifiers are used to control different levels of air humidity. (E) Resistance response caused by different power levels of the humidifier.

With the advancement of artificial intelligence technology, environmental recognition technology has reached a new stage. With the help of artificial intelligence technology, robots can recognize obstacles in the environment and plan their own paths with precision. However, relying solely on single image recognition technology is not enough to fully understand the environmental conditions in which a robot operates. For example, there could be colorless and odorless gases in the environment that can pose a threat to human beings, such as carbon monoxide. In order for robots to better explore unfamiliar and potentially hazardous territorial environments [31,32], specific sensors are essential. McKeown et al. [33] have successfully coated the cantilever beam sensor with metallic palladium to achieve the sensing and measurement of hydrogen gas. As a kind of biomimetic sensor, BTHMs need to perceive changes in the external environment, such as humidity and hardness, similar to the tactile sensing systems found in animals. Here, we have combined superabsorbent hydrogel with BTHM to create a new type of sensor for detecting air humidity, as shown in Fig. 7A and B. It is worth mentioning that superabsorbent hydrogel has a huge range of mass change (Fig. 7C). To better secure the superabsorbent hydrogel microspheres on the rod of the BTHM, high adhesive glue is used for the connection. During testing, the substrate of the humidity sensor is either adhered or mechanically fixed to the external environment (Fig. 7D). By using a humidifier, the humidity sensor can detect the moisture content in the air. When the power of the humidifier increases, the moisture content in the air increases as well. As a result, the superabsorbent hydrogel expands, causing deflection in the stem of the sensor. Consequently, the attached sensing layer on the base will experience significant resistance changes due to the bending of the base. We set the same testing time of 10 min to verify the relationship between the power levels of the humidity sensor and the humidifier. It can be observed that the higher the power of the humidifier, the greater the rate of change in resistance of the humidity sensor, as shown in Fig. 7E. Based on this, the humidity sensor has established a good foundation for robots to explore complex and unknown environments.

## Conclusion

Sensors have already been involved in various aspects of human life and have been widely applied in various fields. Therefore, it is of great significance to improve the performance of existing sensors and explore the design principles and fabrication processes of the next-generation sensors. As a typical representative of carnivorous plants, the sensory hairs of Venus flytrap perfectly achieve the organic combination of high perceptual accuracy and microscale features. Therefore, exploring the excellent performance of Venus flytrap sensory hairs and conducting corresponding biomimetic research have provided inspiration for the development of novel sensors. We were inspired by the unique cantilever beam structure of the trigger hair of Venus flytrap. As a result, we developed a bio-inspired strategy to enhance the proprioceptive capability of smart devices by utilizing the cantilever beam structure for the development of mechanosensors. The BTHM mainly consists of a rigid rod and a flexible base rod with a piezoresistor on the notch structure. Analogous to biological sensilla, a variable stiffness cantilever beam structure mechanosensor can easily realize strain concentration under loading due to the ingenious “notch structure” design. Analogous to biological sensilla, the flexible base rod would experience bending deformation as the rigid rod deflection. The BTHM met all the requirements for mechanosensors, such as mechanical stability, repeatability, and response to mechanical signals. In addition, we have explored a series of application tests, including the detection of micro load, air flow, and vibration, which showed excellent detection performance for the dynamic signal caused by object. The study presents an appealing biologically inspired strategy. This strategy involves using the cantilever beam structure to amplify signals and maximize the sensing performance of mechanosensors. The facile fabrication and excellent sensing performance of the BTHM have paved a way for future applications in mechanosensors. However, the processability should be optimized in future work to enable large-scale fabrication.

## Data Availability

The authors declare that the data supporting the findings of this study are available within the paper. Should any raw data files be needed in another format, they are available from the corresponding author upon reasonable request.

## Supplementary Materials

Supplementary 1 Fig. S1. Preparation steps and related instruments for BTHM bases with notched structure. Fig. S2. Testing platform built to systematically characterize the performance of BTHM. Movie S1. The preparation of a notch structure of a capillary glass tube using a microelectrode drawing instrument. Movie S2. Different shapes of the basal podium and the hair lever of trigger hair of Venus flytrap under external stimulation.
