# Supplementary material for: Mechanosensor for Proprioception Inspired by Ultrasensitive Trigger Hairs of Venus Flytrap
Source: Cyborg Bionic Syst. 2024 Jan 24;5:0065. doi: 10.34133/cbsystems.0065 (PMC10807870; doi:10.34133/cbsystems.0065)
Supplement: Supplementary 1 — Fig. S1. Preparation steps and related instruments for BTHM bases with notched structure. Fig. S2. Testing platform built to systematically characterize the performance of BTHM. Movie S1. The preparation of a notch structure of a capillary glass tube using a microelectrode drawing instrument. Movie S2. Different shapes of the basal podium and the hair lever of trigger hair of Venus flytrap under external stimulation. [file cbsystems.0065.f1.zip › Supporting Information.docx]

**Supplementary Information**

**Mechano-Sensor for proprioception inspired by ultrasensitive trigger hairs of Venus flytrap**

Qian Wang, Zezhong Lu, Deshan wang, Kejun Wang*

Jiangsu Provincial Key Laboratory of Advanced Robotics, Soochow University, Suzhou 215021, P.R.China

*Kejun Wang

kjwang@suda.edu.cn

**Mechano-Sensor for proprioception inspired by ultrasensitive trigger hairs of Venus flytrap**

Qian Wang, Zezhong Lu, Deshan wang, Kejun Wang*

**The word file includes:**

**Figure S1:** Preparation steps and related instruments for BTHM bases with notched structure.

**Figure S2:** Testing platform built to systematically characterize the performance of BTHM.

**Movie S1:** The preparation of a notch structure of a capillary glass tube using a microelectrode drawing instrument.

**Movie S2:** Different shapes of the basal podium and the hair lever of trigger hair of Venus flytrap under external stimulation.


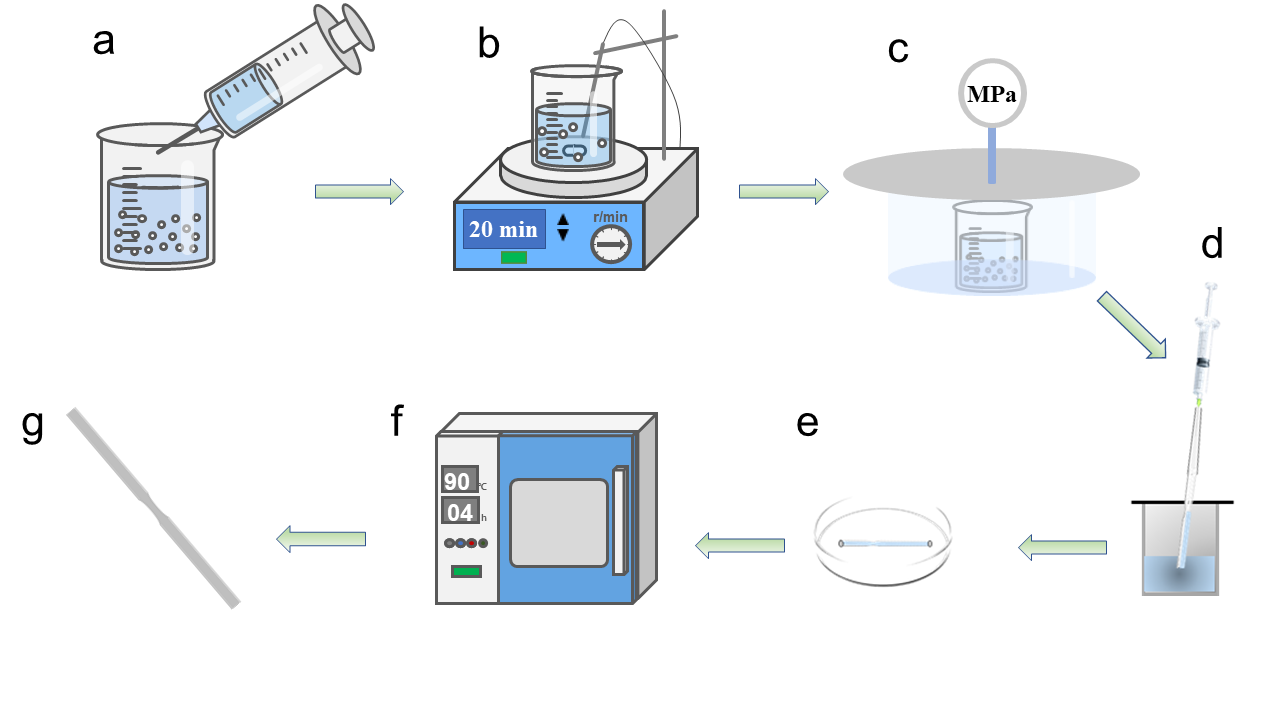


**Figure S1.** Preparation steps and related instruments for BTHM bases with notched structure. (a)The preparation process of PDMS solution. (b) Use a magnetic stirrer to stir the PDMS solution for 20 minutes. (c) Eliminating bubbles in PDMS solution under vacuum. (d) Transfer the PDMS solution to the capillary glass tube mold without introducing any bubbles. (e) Sealed capillary glass tube containing PDMS solution. (f) Place the capillary glass tube containing PDMS in a 90°C drying oven and heat it for 4 hours. (g) Remove the PDMS substrate from the capillary glass tube mold by peeling it off.


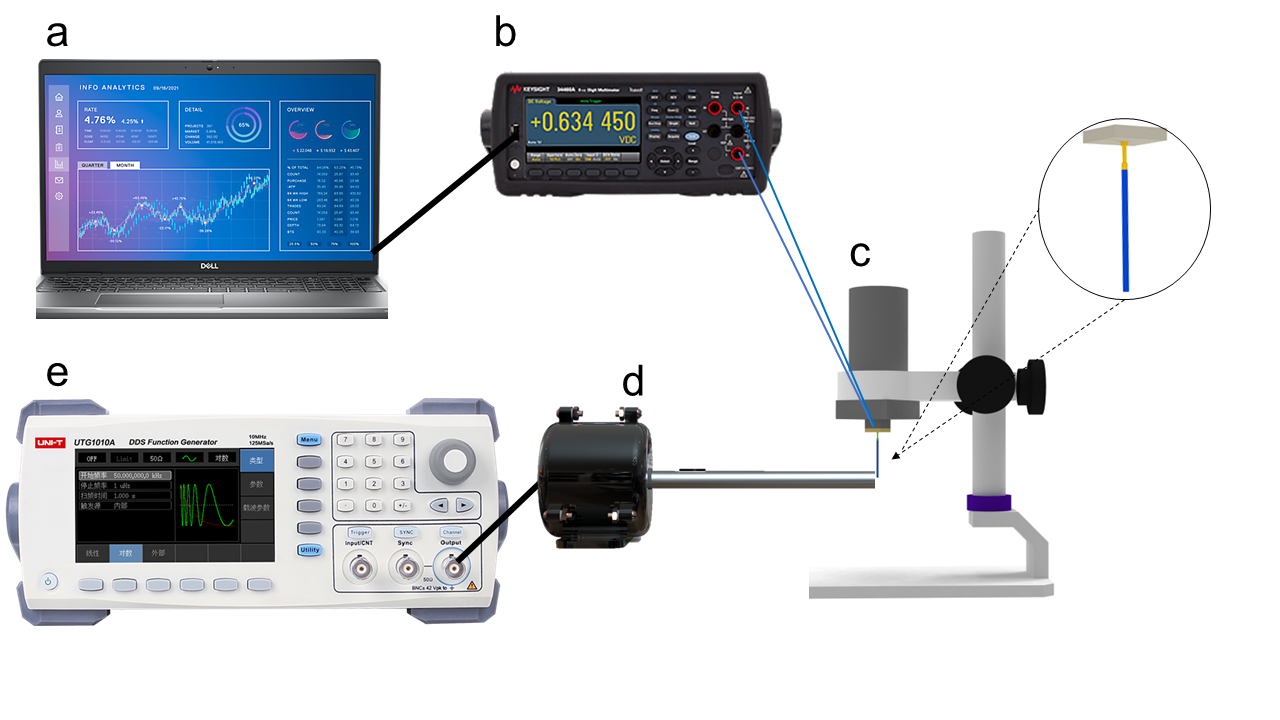


**Figure S2.** Testing platform built to systematically characterize the performance of BTHM. (a) Computer equipped with data analysis software. (b) Digital multimeter to detect changes in BTHM resistance (c) Bracket, used to secure the BTHM. (d) Exciter, applying different stimulus signals to the BTHM. (e) Oscilloscope, providing different waveforms and excitation frequencies.
