# Supplementary figures and images for: Mechanosensor for Proprioception Inspired by Ultrasensitive Trigger Hairs of Venus Flytrap
Source: Cyborg Bionic Syst. 2024 Jan 24;5:0065. doi: 10.34133/cbsystems.0065 (PMC10807870; doi:10.34133/cbsystems.0065)

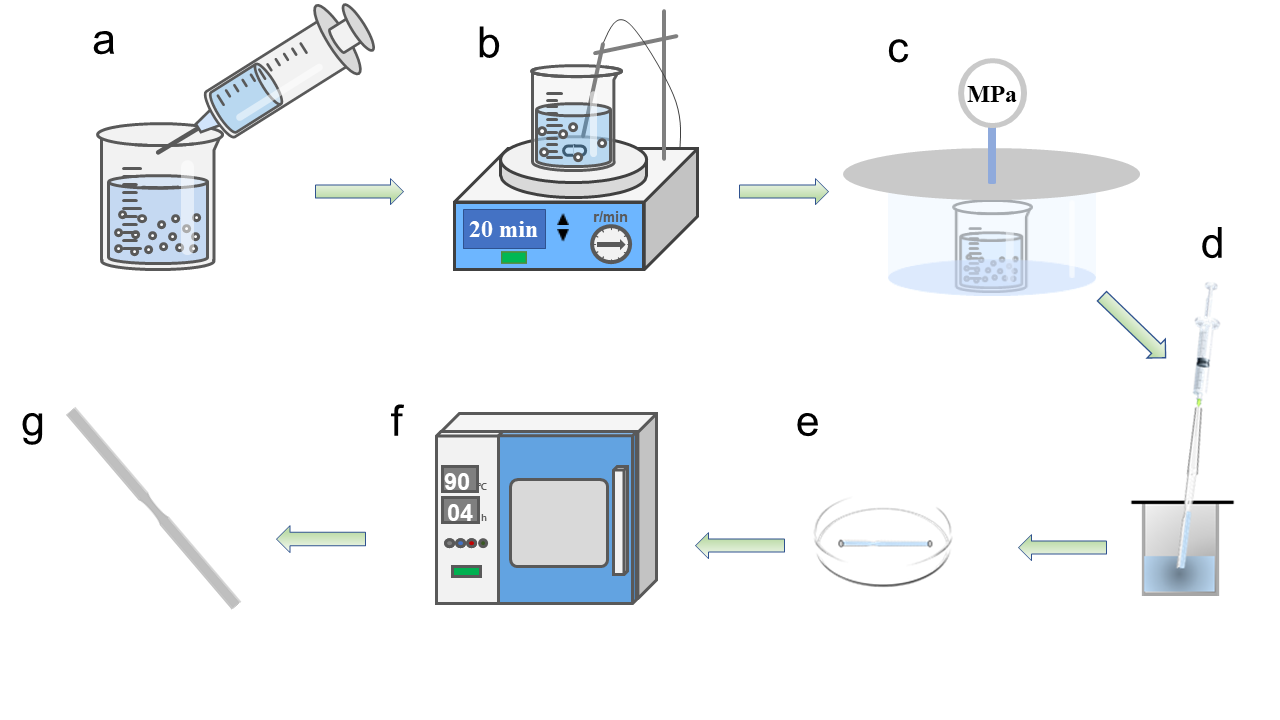

Supplement: Supplementary 1 — Fig. S1. Preparation steps and related instruments for BTHM bases with notched structure. Fig. S2. Testing platform built to systematically characterize the performance of BTHM. Movie S1. The preparation of a notch structure of a capillary glass tube using a microelectrode drawing instrument. Movie S2. Different shapes of the basal podium and the hair lever of trigger hair of Venus flytrap under external stimulation. [file cbsystems.0065.f1.zip › Figure S1.PNG]

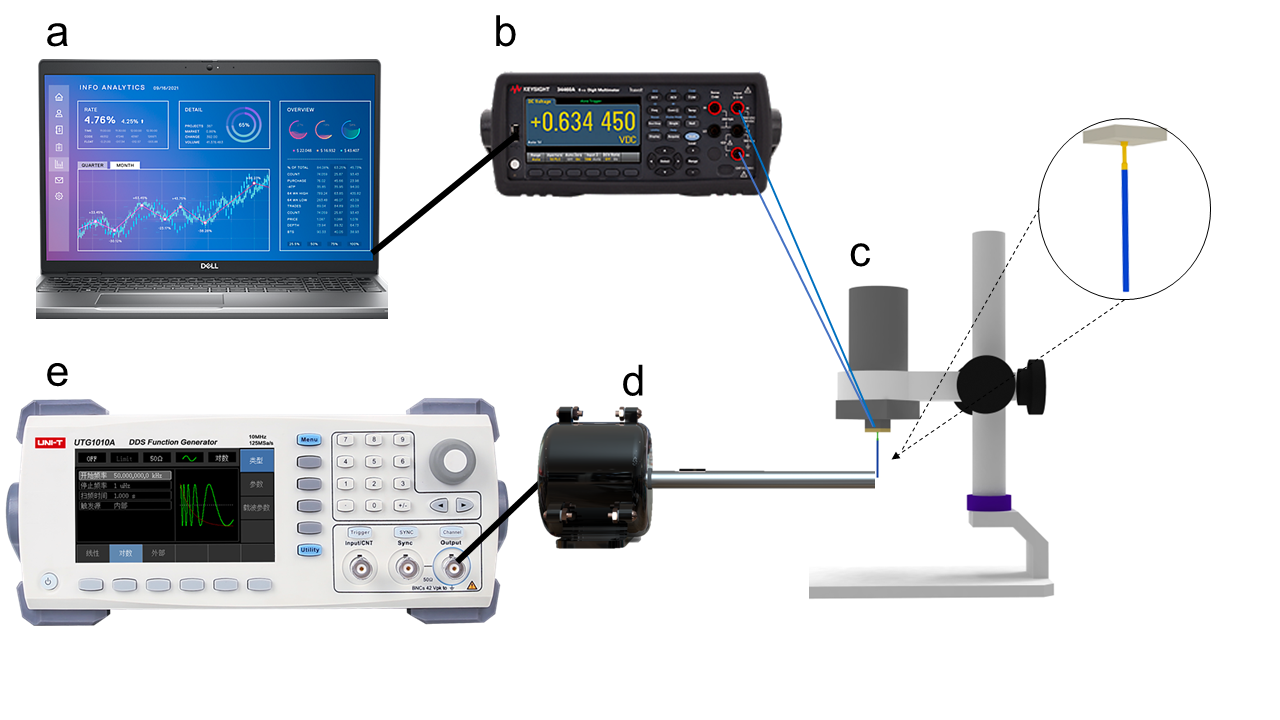

Supplement: Supplementary 1 — Fig. S1. Preparation steps and related instruments for BTHM bases with notched structure. Fig. S2. Testing platform built to systematically characterize the performance of BTHM. Movie S1. The preparation of a notch structure of a capillary glass tube using a microelectrode drawing instrument. Movie S2. Different shapes of the basal podium and the hair lever of trigger hair of Venus flytrap under external stimulation. [file cbsystems.0065.f1.zip › Figure S2.PNG]
